# The Safety of the Kahook Dual Blade in the Surgical Treatment of Glaucoma

**DOI:** 10.7759/cureus.6682

**Published:** 2020-01-16

**Authors:** Mann Barry, Mubark W Alahmadi, Mohammed Alahmadi, Ali AlMuzaini, Mohammed AlMohammadi

**Affiliations:** 1 Ophthalmology, Taibah University, Madina, SAU; 2 Ophthalmology, Tiabah University, Madina, SAU

**Keywords:** ophthalmology, intraocular pressure, saudi arabia, madina

## Abstract

Abstract

Intraocular pressure defects remain a significant problem in the medical field. Several methods have been employed to help reduce the effect of intraocular pressure (IOP), all of which have their merits and demerits. Considered in this paper are surgery and the Kahook Dual Blade (KDB; Kahook, New World Medical, Inc., Rancho Cucamonga, CA) process invented and tested during previous studies to reduce IOP. This study examines both methods; first, in combination with cataract surgery, and then, separately, to assess their effectiveness in reducing IOP.

Objective

The focus of the research is to establish the effectiveness and safety associated with goniotomy while utilizing the Kahook Dual Blade (KDB) procedure for reducing IOP and bringing down the reliance on hypotensive agents in a variety of glaucoma types, either independently or when combined with phacoemulsification (phaco) within the follow-up duration (four to seven months).

Methods and design

The setting was the outpatient clinics in Al-Maghrabi Hospital in Medina. It involved the use of a retrospective study for the study design. Fifty eyes from a total of 45 patients were reviewed. There was KDB in 10 eyes while KDB was integrated with the cataract surgical procedure in 40 eyes.

The de-identified clinical data have been collected by data collectors, who took into account IOP measurements both postoperatively and preoperatively, types of treatment used, side effects, and whether or not there was additional surgery required during the four to seven months observation period.

Results

Seventy percent of the cases in this research had primary open-angle glaucoma. Additional diagnoses were close-angle, normal-tension, pseudoexfoliative glaucoma, and pigmentary. There was a decline in the value of the mean baseline IOP from the earlier 20.66 mmHg ± 7.89 (SD) to a staggering 14.66 ± 3.9 mmHg within the postoperative period (four to seven months) for all 50 eyes, and there was a reduction in hypotensive medication use from 1.54 ± 1.26 to 0.22 ± 0.51 (SD) (P<0.05 and P = .005) for all 50 eyes. In the KDB + phaco group, the mean baseline IOP decreased from 20.75 ± 8.1 (SD) to 13.8 ± 3.7 (P < 0.05 and P = .000), respectively, and the medications used decreased from 1.32 ± 1.18 (SD) to 0.22 ± 0.48 (SD), whereas in KDB goniotomy alone, the mean baseline IOP decreased from 20.30 ± 7.3 (SD) to 18.0 ± 3.0 (SD) (P< 0.05 and P = 0.00) and there was a reduction in the amount of medications from 2.4 ± 1.26 (SD) to 0.2 ± 0.63 (P< 0.05 and P = 0.000).

Eye irritation occurred the most in the KDB + Phaco group, representing 8%, which is resolved within 24 hours postoperatively. As for the KDB only group, hazy vision occurred the most, representing 22% but subsided between two to five days for all patients.

Conclusion

Affirming the results of the study, the safety and effectiveness of goniotomy with the KDB procedure is certain towards the reduction of IOP and medication usage, either independently or when combined with phacoemulsification during four to seven months of follow-up.

## Introduction

Glaucoma is progressive optic neuropathy, and it has been attributed to visual loss that cannot be reversed globally. Reducing intraocular pressure (IOP) has been shown to be the most effective way of stopping the progression for most patients. The number of glaucoma sufferers is expected to increase, along with the aging population [1].

Currently, glaucoma treatment includes ocular hypotensive agents, mainly, laser, trabeculoplasty, and surgical filtration. The major areas that resist aqueous flow include the trabecular meshwork, specifically, the juxtacanalicular part neighboring the Schlemm canal, together with further distal outflow edifices [1-3]. The mechanism for this is still speculative, but the complication could be linked with age-associated changes in the trabecular meshwork. Theoretically, there should be a reduction in resistance by the incision or removal of the trabecular meshwork, bringing about improved control of the IOP [2]. Removing tissue completely may give room to closure resistance by an opening from a surgical procedure, thereby greatly reducing IOP, in place of an incision via the trabecular meshwork [4]. According to the findings of preclinical studies, it is possible to reach almost complete elimination of the trabecular meshwork minus bringing destruction to the close tissues using a single-use dual-blade (Kahook, New World Medical, Inc., Rancho Cucamonga, CA) [5].

The study provides validation of clinical statistics concerning the effectiveness and the added safety of the KDB procedure alone and when it is combined with cataract surgery (phacoemulsification or phaco).

## Materials and methods

Study design

This study involved 50 adult patients’ eyes that underwent treatment for glaucoma through the KDB surgical procedure. Out of the 50 eyes, KDB was used only for 10 eyes while KDB combined with phacoemulsification was used for the remaining 40 eyes. The indicator that was used for goniotomy was decreased IOP or a reduction in IOP-lowering treatments, while also preventing the development of a filtering bleb as well as its connected threat profile.

Patient data and baseline were composed first preoperatively on the same day the surgery was to be done and then in three intervals - the first one (1-10 days), the second (1-2 months), and the third (4-7 months) postoperatively. Data composed of the severity and form of glaucoma, IOP, used medication, operative details, complications, and postoperative prognosis. We collected the personal identifying data, including the demographic data in this identified data set.

Single-use dual blade

The design of the Kahook single-use dual blade was for the smooth access of the blade’s sharp distal tip into the trabecular meshwork and Schlemm canal. Before pushing the device through the trabecular meshwork, there is the correct setting of the blade against the canal’s anterior wall. A ramp moves up from the distal tip, followed by the elevation of the trabecular meshwork tissue overhead its location, and the tissue guided headed for two blades. After this, there is the creation of analogous slits in the trabecular meshwork, using the blades for the elimination of the trabecular meshwork strip that is not affected. The manufacturer recommends cutting the tissue perfectly, with minimal damage to the nearby structures. It is necessary to lift the trabecular meshwork overhead its normal location and stretch it prior to making the cut. The distal cutting surface angle, as well as the device shaft size, was designed such that the highest angle treatment is possible via a single clear corneal incision (CCI). Despite its initial design for the treatment of glaucoma by the removal of the trabecular meshwork in the eye that needs a goniotomy, it is also possible to use the single-use dual blade for supplementary intraocular procedures like extracting a cataract in a combination cataract extraction.

Surgical technique

The following describes the part of the procedure that involved the use of the single-use dual blade. The insertion of the device was through the temporal CCI utilized to place phacoemulsification and IOL. The anterior chamber was protected using a supplementary ophthalmic viscosurgical device (OVD) when needed. The rotation of the head of the patient was between 30 and 45 degrees in the opposite direction of the doctor, whereas the tilting of the microscope was between 30 and 45 degrees along with the doctor’s position. This was followed by the placement of a straight gonioprism on the cornea, and the non-dominant hand and the anatomic standards, together with the trabecular meshwork, coming into the center. The insertion of the dual blade along the CCI followed. The piercing of the trabecular meshwork was carried out using the dual blade tip with the footplate heel sitting against the Schlemm canal’s anterior wall. The pushing of the device through the Schlemm canal was either in a clockwise or a counter-clockwise bearing. The trabecular meshwork was adequately removed in one bearing, and the dual blade was revolved to about 180 degrees while the dual blade’s tip was positioned at some clock hours away from where the first treatment took place. Another push of the device was for the creation of a trabecular meshwork strip that floats freely for the visibility of Schlemm canal’s back, such that the previously treated trabecular meshwork can be reached. In the end, the dual blade from the eye was removed, followed by the free-floating trabecular meshwork by aspiration or intraocular pincers with the irrigation-aspiration handpiece while the OVD was being removed.

Postoperative medications and follow-up

The selected protocol of the surgeon was used to manage every patient postoperatively, and it was relatively that of Al-Magrabi Hospital. This included the use of a prednisolone acetate 1%, topical fourth-generation fluoroquinolone, and nonsteroidal anti-inflammatory drugs. Topical drops were given as the preferred form of medication, but intracameral moxifloxacin 0.5 mg/0.1 mL was used for endophthalmitis prophylaxis in all cases. Because this was a retrospective study, the removing and adding of these medications were dependent on clinical judgment with the object of reaching a target IOP according to the patient’s clinical condition.

Result measures

The primary assessor of outcome in the research depended on the use of Goldmann applanation tonometry to measure the IOP, and on the ratio of decline in the patients’ IOP being over 20% from the reference line. The secondary outcome measure encompassed the quantity of ocular hypotensive medications and the ratio of patients with the schedule that reduced by over one medication. The procedure safety was assessed by the use of expressive exploration of unfavorable happenings noted by the physician, from the patient’s report, or both; this involved considerations of the necessity of reoperation or otherwise.

The assessment of the surgeons’ experience in the procedure was done by asking about their perspective (strongly agree, or undecided/neutral, or disagree, or strongly disagree) concerning the statements that follow:

i. The performance of goniotomy utilizing the single-use dual blade was simple.

ii. Entering into the Schlemm canal using a single-use dual blade was simple.

iii. The efficiency in the progress of the single-use dual blade after entry into the Schlemm canal.

Numerical assessment

SPSS statistics software (version 24.0, IBM Corp., Armonk, NY) was used to perform statistical analysis.

## Results

Fifty patient’s eyes data was used for this study. Forty eyes underwent KDB with the phacoemulsification cataract surgical procedure and 10 eyes had KDB alone. It involved surgeons with experience in angle-based surgery. Table 1 shows the mean IOP difference between preoperative and postoperative on each visit for all 50 eyes with KDB alone and (phaco + KDB). The mean IOP value decrease from 20.66 ± 7.89 mmHg preoperative to 14.78 ± 5.96 mmHg, 17.4 ± 7.6 mmHg, 14.66 ± 3.9 mmHg at one to 10 days, one to two months, four to seven months follow-up, respectively, with P-value = 0.00 for all eyes. Table 2 shows the mean comparison between the mean numbers of medications preoperatively and postoperatively for all patients. There was a significant decrement in the mean IOP and the mean number of hypotensive treatments from the reference point to four to seven months postoperatively (P < .001 and P = .005).

**Table 1:** Mean intraocular pressure (IOP) different preoperative and postoperative for 50 eyes

	IOP	Mean (SD)	P-value
Preoperative intraocular pressure (IOP)	1 day before	20.66(7.89)	0.00
Postoperative IOP	IOP (1-10 days)	14.78(5.96)	
	IOP (1-2month)	17.4(7.648)	
	IOP (4-7month)	14.66(3.9)	

**Table 2:** Average number of medications prior to and after the operation

Pair 1	SD	P-value
Preoperative Drug	1.54(1.265)	.005
Postoperative Drug	0.22(0.507)	

Table 3 shows a comparison between the KDB + phaco group and the KDB only group in the IOP difference and medication use before and after surgery. The mean IOP value decreased from 20.75 ± 8.15 preoperative to 14.15 ± 5.89, 17.52 ± 7.8, and 13,82 ± 3.65 at one to 10 days, one to two months, and four to seven months respectively in the KDB + phaco group, P-value=0.00, and from 20.3 ± 7.3 preoperative to 17.3 ± 5.85, 16.9 ± 7.37, and 18 ± 3.09 at one to 10 days, one to two months, and four to seven months, respectively, in the KDB only group, P-value= 0.00. The medication use decreased from 1.32 ± 1.18 to 0.22 ± 0.48 in KDB + phaco group, P-value=0.005, and from 2.4 ± 1.26 to 0.2 ± 0.63 in the KDB only group, P-value=0.000.

**Table 3:** Comparison of the IOP difference and drug quantity before and after surgery between the two groups intraocular pressure (IOP)

	Mean(SD)	Number (n)	P-value
Kabook Dual Blade with phacoemulsification			
IOP preoperative	20.75(8.12)	40	0.00
Postoperative intraocular pressure (IOP) (1-10 days)	14.15(5.89)		
Postoperative intraocular pressure (IOP) (1-2 months)	17.52(7.8)		
Postoperative intraocular pressure (IOP) (4-7months)	13.82(3.65)		
Drug before	1.32(1.18)		0.005
Drug after	0.22(0.48)		
Kahook Dual Blade (KDB) alone			
Preoperative intraocular pressure (IOP)	20.3(7.3)	10	0.00
Postoperative intraocular pressure( IOP) (1-10days)	17.3(5.85)		
Postoperative intraocular pressure( IOP) (1-2months)	16.9(7.37)		
Postoperative intraocular pressure( IOP) (4-7months)	18(3.09)		
Drug before	2.40(1.26)		0.000
Drug after	0.2(0.63)		

**Figure 1:**
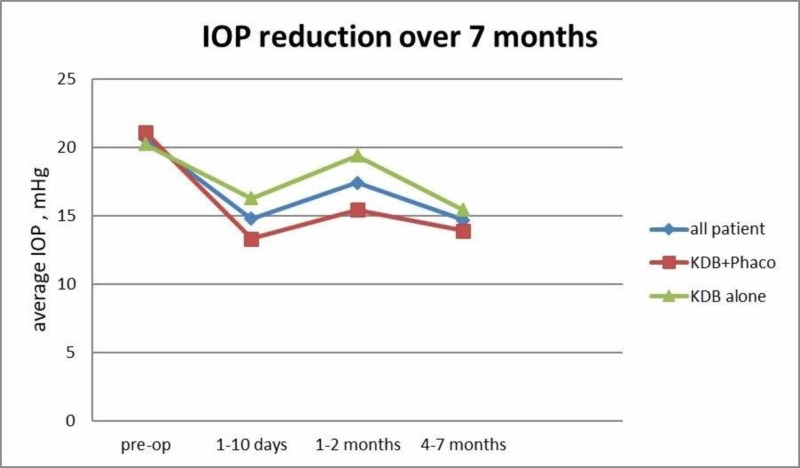
Timeline of intraocular pressure (IOP) reduction for all patients, KDB+Phaco and KDB alone group over 7 months

Figure 1 shows the timeline of the mean IOP difference before and after surgery for both groups, KDB standalone and KDB + phaco over four to seven months follow-up.

We conclude from the results in Table 3 and Figure 1 that combined cataract surgery with KDB has a higher chance of decreasing the IOP.

**Figure 2:**
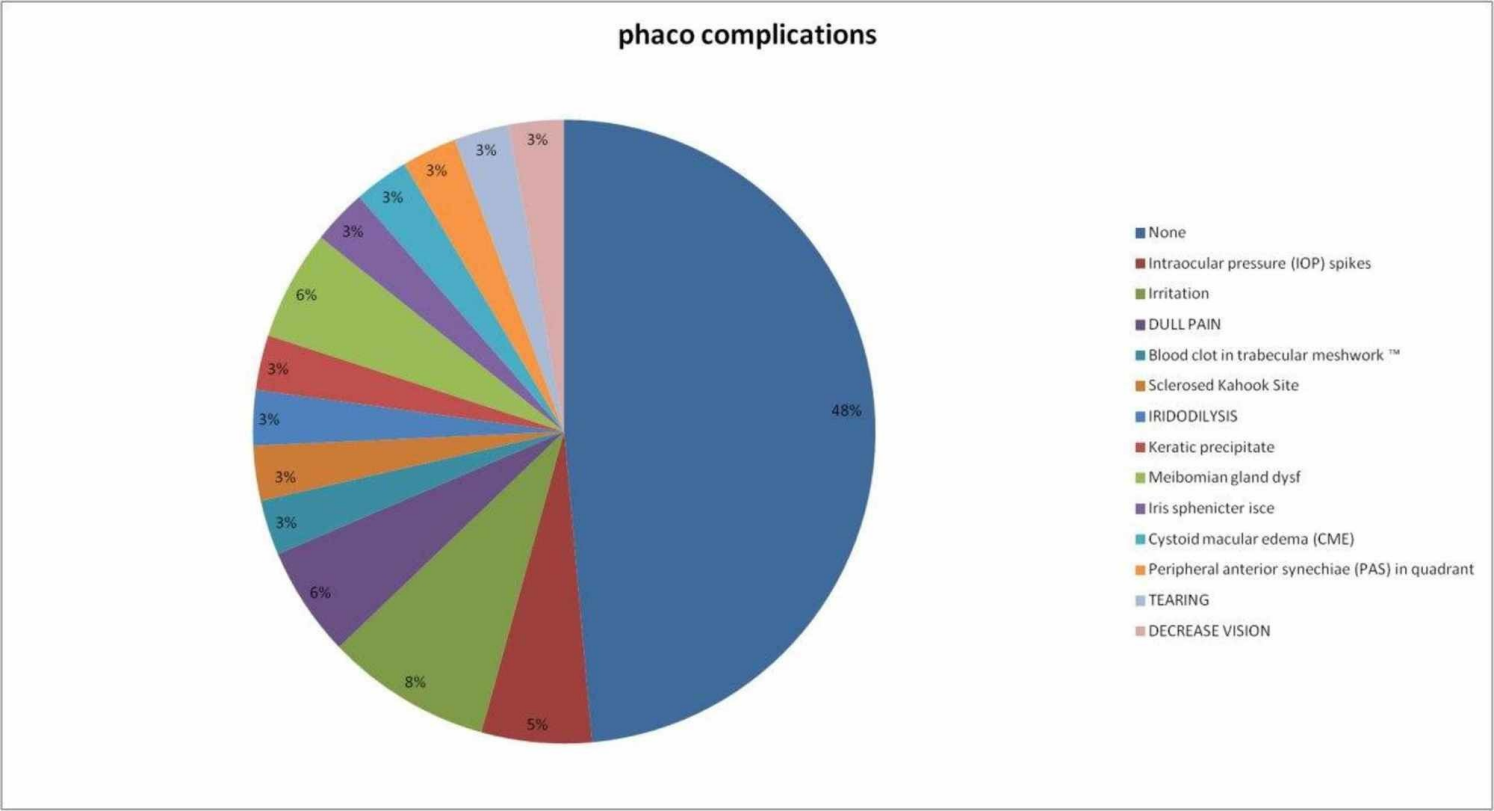
Frequency circulation of Kahook Dual Blade + phaco complications

The most common complication in the KDB + phaco group was eye irritation by 8%, which disappears within 24 hours postoperatively, while those who had no complications were 48%, as presented in Figure 2.

**Figure 3:**
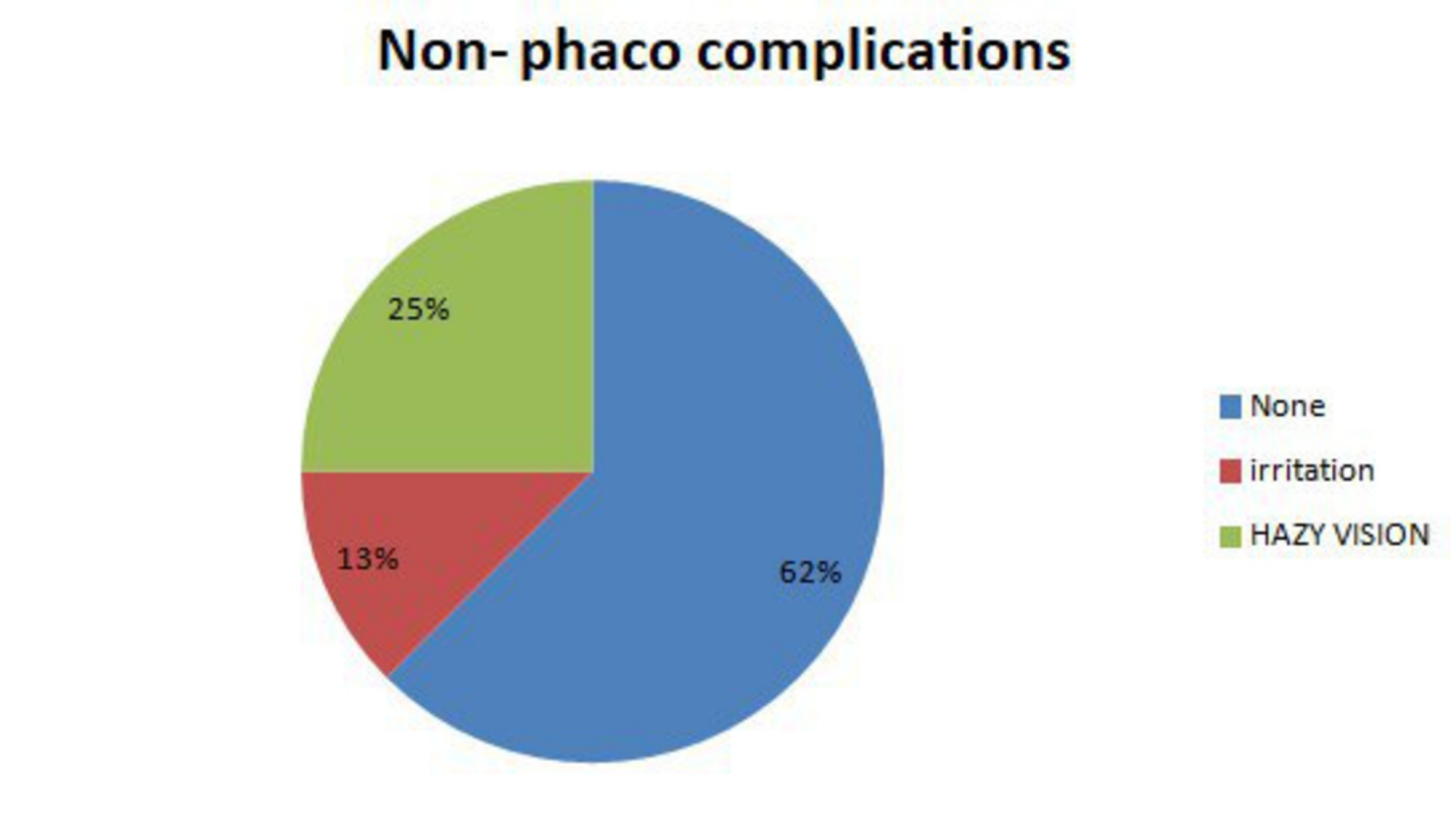
Kahook Dual Blade alone complications frequency distribution

Figure 3 shows the frequency distribution of complications in the Kahook Dual Blade alone group.

Table 4 demonstrates the complication for 50 eyes for both groups, hazy vision, and IOP spike were the most common complication with 17.1 % for both of them. The hazy vision disappears within two to five days and the IOP spike disappears within 24 hours postoperatively.

**Table 4:** Frequency distribution of complication in patients with reduced IOP of 20% or more from baseline intraocular pressure (IOP)

Group Complications	Frequency	Percent
irritation	4	11.4
Kahook effect on angle 2 quad oculus sinister (OS)	1	2.9
Intraocular pressure (IOP) spike	6	17.1
Posterior capsular opacification (PCO)	2	5.7
Cystoid macular edema (CME)	2	5.7
Pain	3	8.6
Hazy vision	6	17.1
Difficulty remaining the trabecular meshwork (TM) strip	2	5.7
Blood reflux	1	2.9
Allergic Rhinocongunctivits oculus uterque (OU)	1	2.9
Anterior chamber reformation	1	2.9
A blood clot in the trabecular meshwork (TM)	1	2.9
Meibomian gland dysfunction	3	8.6
Mild redness	1	2.9
Sclerotedkhook site	1	2.9

## Discussion

The demonstration of the efficiency and safety of the KDB standalone procedure and when the KDB procedure and cataract surgery are combined, using clinical data, was the goal of this study. Patients who underwent surgery had moderate to end-stage glaucoma. The surgery was successfully combined with phaco in 40 eyes and KDB alone in 10 eyes. KDB recorded a significant lowering of IOP by 29.04% and a decrease in medication by 85.71% in all patients for both groups. The IOP reduction in (KDB + phaco) was 33.39%, and medications reduced by 83.33%. Contrarily, in KDB alone, IOP was reduced by 11.33% and there was a decline in the amount of medication by 91.66%. The IOP showed a reduced effect after this procedure and the results were yielded in four to seven months of follow-up for all of these groups.

From our results, it would suffice to say that the IOP gradually reduces with time after the operation as seen in the relatively higher IOP before surgery as compared to IOP after one to 10 days, one to two months, and four to seven months (P=0.000) for all patients. The efficiency of KDB and cataract surgery in reducing IOP was observed, as was the case with Sieck (2018), in their investigation of the outcomes of KDB and phacoemulsification [6]. Due to the effectiveness of surgery, medication use was significantly lower after surgery (P-value=0.005), in the KDB+phaco group. Also, in the KDB only group, the decrease in the number of medications used was significant (P-value=0.00). This finding was also the same in the study by Dorairaj (2018), where they emphasized that the method with the greatest effectiveness to reduce IOP was surgery [7]. However, the method also gives rise to other complications. An interesting finding from the present study was that the remarkable decline in the number of drugs used before and after surgery in both KDB alone and KDB with phacoemulsification (P<.05 in both cases), there was also a significant decrease in the IOP in KDB + phaco from 20.75 (8.12) to 13.82 (5.85) over four to seven months (P<.05). Whereas the decrease in IOP value in KDB alone was from20.3 (7.3) to 18 (3.09) during four to seven months (P<.05). Overall, combined KDB plus cataract surgery has a great decrease in IOP measurement postoperatively. This finding again confirms the finding by Dorairaj (2018), which emphasizes the effectiveness of surgery in the reduction of IOP [7]. As evidenced in the findings of the present study, IOP reduced with time since surgery [8]. The low IOP effect may be due to the removal of most of the trabecular meshwork with protection against the destruction of nearby structures.

The fact that it is possible to do KDB while cataract surgery is ongoing or alone is a merit of KDB. Other ab internal-glaucoma procedures can only be carried out when cataract surgery is ongoing. Therefore, as KDB was successful with or without phaco, KDB goniotomy can be used to treat phakic and treat former pseudophakic patients. Apart from the IOP decrement, minimal complications were observed in our study results, in line with those of Mizoguchi [9]. Hazy vision was the most common complication, which occurred by 25% in the KDB only group and disappeared within two to five days for all patients, and in KDB + phaco, the most common complication was eye irritation by 8% and resolved within 24 hours.

The lack of randomization of patients, which rendered the study quasi-experimental, could be pointed out as one of the restrictions of the present study. Quasi-experimental designs are powerful methods of sample and population analysis, but they suffer from poor internal validity where it is hard to determine whether the effect is really due to treatment. While the t-test is a powerful method for pairwise comparison in small samples, having a sample bigger than 10 in KDB alone would have increased the magnitude of the effect and hence the external validity of the results. Finally, there was a limitation of follow-up for the four to seven months duration. Our study will remain to be monitored for the enhanced assessment of the IOP decreasing impact at each time point and past the seven months duration, as the follow-up phase continues to amass.

## Conclusions

KDB goniotomy as an ab interno procedure can be effectively and safely used to reduce IOP and the number of medications in patients with severe glaucoma. The profile of the procedure’s safety is outstanding and has consistency with other ab interno procedures either in combination with cataract surgery or alone. Furthermore, the complication rate is low and most of the complications were minors and reversible within a few days.
